# Psychometric properties of the Quiet Ego Scale (iQES) within the Italian cultural context

**DOI:** 10.3389/fpsyg.2024.1348965

**Published:** 2024-05-09

**Authors:** Laura Bernabei, Cristiana Silvestrini, Lucrezia Andrea Grimaldi, Valentina Piserchia, Francesco Riggio, Giovanni Marino, Donatella Valente, Enrico Pompili

**Affiliations:** ^1^Human Neuroscience Department, Sapienza University of Rome, Rome, Italy; ^2^Psychiatric Service of Diagnosis and Treatment, Mental Health Department and Addiction, Azienda Sanitaria Locale Roma 5, Colleferro, Italy; ^3^Mental Health Centre Tivoli, Mental Health Department and Addiction, Azienda Sanitaria Locale Roma 5, Tivoli, Italy; ^4^Mental Health Centre, Mental Health Department and Addiction, Azienda Sanitaria Locale Roma 5, Colleferro, Italy

**Keywords:** quiet ego, self-compassion, psychometric properties, self-esteem, wellbeing, mindfulness, personal growth

## Abstract

**Introduction:**

The quiet ego indicates a more compassionate conception of self-identity that integrates others into the self by lowering the intensity of the ego and enhancing the awareness of the present moment. The Quiet Ego Scale (QES) is a 14-item self-report measure of quiet ego, and it is composed of the following four psychological domains: detached awareness, inclusive identity, perspective taking, and growth. The present study aimed to test the psychometric properties of the Quiet Ego Scale within the Italian cultural context (iQES).

**Methods:**

A total of 160 Italian university students aged between 20 and 42 years, with a mean age of 22.85 years (SD = 3.41), completed the measures of the iQES and of other psychological dimensions. The psychometrics properties of iQES were assessed based on its internal consistency, test-retest reliability, and construct validity through comparisons with other correlated psychological measures.

**Results:**

Analyses confirmed the psychometrics properties of iQES. As in previous studies, the quiet ego was positively associated with the indicators of resilience (*p* < 0.01), happiness (*p* < 0.05), self-esteem (*p* < 0.01), and psychological wellbeing (*p* < 0.01).

**Discussion:**

The study discussed the possible uses of the iQES in the field of mental health, specifically focusing on improving adherence to psychological therapies and enhancing psychological and social well-being. The results indicated strong psychometric properties of the iQES in measuring the quiet ego construct. Our findings enrich the literature on the validity of the iQES and highlight the multidimensional nature of the quiet ego construct.

## Introduction

The “quiet ego” is a multifaceted concept developed in contemporary psychology and philosophy (Wayment et al., 2015a). It aims to identify the subjective position that an individual can assume toward themselves and others, which is characterized by reduced volume, deep listening to oneself and others, modesty, tranquil awareness, and lesser concern for the ego. The quiet ego approach to life is focused on the idea of being more self-aware and less ego-driven.

The quiet ego has been proposed as an alternative to the traditional view of the ego as dominant, egocentric, and self-centered, which allows us to approach life more humanely while maintaining healthy psychological functioning that stems from stable and secure self-esteem (Akca and Sumer, 2016).

A quiet ego is also defined as an orientation toward the world and the self that reflects lesser emphasis on self-promotion and greater openness to others and the surrounding world, thereby potentially leading to greater happiness, satisfaction, and emotional wellbeing, as it is associated with the reduced pursuit of personal gratification and increased connection with others (Wayment and Bauer, 2018).

The concept of quiet ego is highly intricate as it reflects the relationship between an individual's inner self and the external world in terms of life satisfaction and wellbeing. Bauer and Wayment (2008) suggested that the quiet ego, adopting an organismic perspective, considers the self and others within the context of a broader conception of the individual, orienting toward significant life traits, such as wisdom and virtues, thereby enabling the pursuit of a good life. The qualities of a quiet ego are extremely broad, akin to a list of virtues, which include forgiveness, gratitude, humility, generativity, altruism, interdependence, self-compassion, compassionate love, and much more.

However, the authors have identified four main characteristics of the quiet ego: (1) detached awareness can be understood as a non-critical approach to processing information and observing a situation in an objective and impartial manner. This concept aligns with mindfulness; (2) inclusive identity refers to the extent to which one identifies with others and considers oneself equal to others. For its qualities, it increases the likelihood of cooperation; (3) perspective-taking, a prerequisite for cultivating a quiet ego, is defined as taking on the perspectives of others about oneself; and (4) growth is considered as the ultimate step of the quiet ego, as it incorporates all its other characteristics (Wayment et al., 2015a).

According to Bauer and Wayment (2008), growth is understood as a development over time in a humanistic and prosocial manner. Individuals who can achieve a certain level of growth reflect on their current life as part of a long journey, rather than viewing the future as an outcome. It involves shifting attention from the immediate moment to long-term humanistic personal growth.

It is important to note that the concept of quiet ego is still a subject to be studied and debated within the academic community, and there are various interpretations and approaches to its understanding and application. Some see it as an ideal to pursue for greater personal fulfillment and wellbeing, while others may be skeptical about its effectiveness or practicability in all situations.

The more compassionate portion of the personal identity is reflected by a quiet ego (Wayment and Bauer, 2018). Moreover, it requires the integration of others into the self, nurturing awareness of the present moment. In this context, Bauer and Weatherbie (2023) argued the parallelism between the concepts of “epicurean ataraxia” and “buddhist upekkha” and quiet ego, since all represent models of a good life, as closer to the concept of human flourishing, thereby emphasizing equanimity. It is possible to conceptualize, in fact, the ego on a one-dimensional continuum, where at one end is the noisy ego, which is so preoccupied with itself that it cannot consider other people's points of view because it is clouded by its own ego, and at the other end is the quiet ego (Akca and Sumer, 2016). The noisy ego would appear to be characterized by a defensive attitude that aims to always protect its privileges, sometimes which is detrimental to others.

The concept of the quiet ego also argues that exaggerated attention to self or others can lead to maladaptive psychosocial outcomes over time (Leary et al., 2006).

In some research studies, the quiet ego has been negatively correlated with the dark triad of personality, a specific personality trait that could explain the presence of some antagonistic behaviors and which refers to the adoption of antagonistic social strategies characterized by high levels of interpersonal manipulation and insensitive behaviors (Kaufman et al., 2019). In their study, Kaufman et al. juxtapose the existence of the dark triad of personality with a light triad characterized by personality traits of openness toward others (defined as Kantianism, humanism, and faith in humanity) and the light triad exhibited a particularly strong positive correlation with the total score of the Quiet Ego Scale, while the dark triad demonstrated a negative correlation. The possibility of identifying all these different personality traits may aid in studying specific aspects of human nature, such as the development of benevolent or malevolent facets of personality.

A recent study highlighted not only the importance of having a compassionate self-identity but also the possibility of increasing this ability to manage daily stressful situations and to improve coping capacities (Gilbert, 2020).

Moreover, contemporary theories and research studies suggest that there may be numerous benefits and uses of the quiet ego (Wayment et al., 2015a; Wayment and Bauer, 2018).

Some studies have shown that the quiet ego is positively correlated with prosocial characteristics, such as authenticity, agreeableness, cooperative thinking, and open-mindedness (Wayment et al., 2015a, 2018). This suggests that a quiet ego can promote social exchanges by facilitating relationships between individuals and favoring their success and that it also increases the likelihood that a person intends their activities to benefit both themselves and others (Wayment et al., 2015a; Wayment and Bauer, 2018).

The concept of the quiet ego has been employed in studies on the perceived cost/benefit of some participants regarding their intention to maintain their participation in prosocial activities, such as volunteering. In the study by Vecina et al. (2023), the quiet ego is considered a characteristic that can be more fully expressed and further developed through active participation in prosocial organizations, almost like in a virtuous circle, where the mutual benefits of sharing reinforce the traits of a quiet ego.

Chew and Ang (2023) demonstrate how, given the reciprocal nature of the self and society, the possibility of developing a personal identity based on the quiet ego and self-compassion could promote a more inclusive, robust, and dynamic culture rooted in kindness and authenticity, thereby resulting in increased life satisfaction. These elements assume even greater relevance in specific life stages, such as adolescence, during which the ability to accept and appreciate one's uniqueness and differences from others facilitates the access to and expression of the true self.

The quiet ego is not conceived as a personality trait but as a set of psychosocial skills and abilities that facilitate personal growth (Wayment et al., 2015a; Wayment and Bauer, 2018; Wayment and Cavolo, 2019).

In fact, some authors suggested that the enrichment of quiet ego characteristics, for example, through specific training, can improve some aspects related to psychosocial outcomes (Kaufman et al., 2019).

However, given the relative novelty of the quiet ego construct, it is unknown whether or how a quiet ego translates across cultures and settings.

The Quiet Ego Scale (QES) developed by Wayment et al. is a 14-item self-report measure of the quiet ego, and it is built on the following four subdomains: inclusive identity, detached awareness, perspective-taking, and growth.

Wayment and Bauer developed a measure of the quiet ego, considering it advantageous for measuring a set of characteristics reflecting a person's propensity to think, feel, and behave in ways that are broadly compassionate. The quiet ego is a relatively abstract concept that cannot be directly measured but rather inferred from what the four characteristics of the quiet ego have in common.

Therefore, considering this conceptualization, as advocated by Wayment et al. (2015a), the best methodology for validating a measure of the quiet ego is direct, by psychological scales that assess each of the quiet ego characteristics.

This study aims to translate the Quiet Ego Scale (iQES) into Italian and investigate its psychometric properties within the Italian culture, as there is currently no scale in this language that assesses the aspects of a quiet ego. Since Wayment et al. (2015a) studied the relationship between the quiet ego and other psychological constructs, a secondary goal is to assess whether certain variables, such as demographic characteristics or life experiences, may characterize the quiet ego's expression in different age groups.

## Materials and methods

### Study design

A sample of 160 Italian university students were assessed with the scales of the quiet ego (iQES) and other psychological dimensions. In the present study, students from the first, second, and third years of various undergraduate programs in healthcare professions (physical therapy, occupational therapy, education, psychiatric rehabilitation technicians, neuropsychomotor therapists of developmental age, etc.) were recruited. During the course of study, these students engage in clinical internships and are involved in therapeutic relational dynamics that solicit various personal and relational components, such as personal wellbeing and growth, resilience, self-esteem, and happiness. The opportunity to investigate these variables alongside the quiet ego assumes great relevance, especially considering possible interventions aimed at improving personal wellbeing.

Since some students enroll in these degree programs after gaining work experience or after choosing different degree paths, we found it useful to divide the sample into two clusters: one characterized by students who immediately chose a degree program in healthcare professions after completing high school (and who may likely demonstrate a greater tendency toward compassion, inclusive identity, and growth) and one that included students who arrived after having other experiences and perhaps felt the need to attune with the other in a different way, perhaps in a more compassionate manner.

The entire process of translating and validating the instrument was carried out according to the main guidelines provided in the literature (International Test Commission, 2017; Tsang et al., 2017), which first involved translating the instrument through forward and backward translation procedures, as detailed in the following paragraph. Subsequently, to ensure that the Italian version of the QES was both valid and reliable, the following steps were undertaken: (i) internal consistency analysis, using Cronbach's alpha; (ii) test-retest reliability (with a subgroup of the sample) using Pearson's *r*, to test the stability across time; (iii) construct validity analysis that can be conducted by assessing the iQES correlation with other variables; and (iv) evaluations of construct validity, using exploratory factor analyses (EFA) and confirmative factor analyses (CFA), to ensure that the translated version accurately reflected the original (Cronbach and Meehl, 1955).

### Instruments

To assess construct validity, measures were selected based on instruments that had been previously validated in Italian and were available for free use as a gold standard. Based on various experiments conducted by Wayment et al. (2015a) and Akca and Sumer (2016); the following measures have been selected.

### Compassionate self-identity

#### Quiet Ego Scale

The Quiet Ego Scale (QES) (Wayment et al., 2015a) is a measure of self-identity and is understood as a personality trait that stimulates the connection with the self and others. The QES is a 14-item self-report, characterized by four different subdomains, which include four psychological characteristics such as detached awareness, inclusive identity, perspective-taking, and growth. Nevertheless, all 14 items are used to create a single score, which are not meant to be divided into sub-scores. All items were assessed on a 5-point scale from 1 (*strongly disagree*) to 5 (*strongly agree*). Items 2, 6, 10, 11, and 14 had a reverse code. A higher total score corresponds to a higher quiet ego.

The Cronbach's alpha for the full scale was 0.76, which is similar to the findings of Wayment et al. (2015a) that found Cronbach's alpha of 0.78.

### Psychological wellbeing

#### Psychological Wellbeing Scales

Ryff's Personal Wellbeing Scale (Ryff, 1989; Ruini et al., 2003; PWB) is an 84-item self-rating tool designed to assess personal wellbeing. It is characterized by six subscales that investigate different domains related to psychological wellbeing. These domains, as identified by Ryff, include (i) self-acceptance, (ii) autonomy, (iii) environmental mastery, (iv) personal growth, (v) purpose in life, and (iv) positive relations. Each item is evaluated using a scale of 6 points (1–6): the first three response items of the 6-point scale were codified “*disagree*”, and the remaining three were codified “*agree*”. Some items were formulated negatively and others positively. For each scale, the score can vary from 0 to 14. Cronbach's alpha for the full scale was 0.86.

### Self-esteem

#### Rosenberg Self-Esteem Scale

Rosenberg's Self Esteem Scale (RSE; Rosenberg, 1965) is a largely used instrument to test self-esteem. The scale was validated into Italian language from Prezza et al. (1997). The 10-items were summed using a 4-point Likert scale (0 = strongly disagree; 3 = strongly agree). Cronbach's alpha for the full scale was 0.88.

### Resilience

#### Connor-Davidson Resilience Scale (CD-RISC10)

The Connor-Davidson Resilience scale (CD-RISC) (Connor and Davidson, 2003) is a 10-item self-report, each rated on a 5-point scale (0–4). Greater resilience is reflected in higher scores. We used the 10-item Connor-Davidson Resilience Scale (CD-RISC-10) (Campbell-Sills and Stein, 2007), and royalties for its use were paid to Jonathan Davidson (Translation by Di Fabio and Palazzeschi, 2012). In this study, Cronbach's alpha was 0.88.

### Happiness

#### Subjective Happiness Scale

The Subjective Happiness Scale (SHS) is a 4-item scale designed to evaluate global subjective happiness. Each item is rated using a 7-point scale (1–7), ranging from “*not a very happy person*” to “*a very happy person”* for the first item; from “*less happy*” to “*happier*” for the second item; from “*for nothing*” to “*really*” for the third and fourth items (Iani et al., 2014). The first two items refer to how the person consider himself/herself in relation to happiness. The last two refer to how the sentence describes the subject (Lyubomirsky and Lepper, 1999). Cronbach's alpha for the scale was 0.63, a low reliability in this sample.

### Translation

Heidi Wayment developed the original Quiet Ego Scale, and the translation process was carried out in accordance with her guidelines. The original questionnaire was converted into Italian by an English Professor and a Senior Clinical Psychologist. They worked independently to produce two translations. One translator was fluent in the original language and had an in-depth knowledge of the culture, and was able to understand the facets of the concept of quiet ego (Beaton et al., 2000). The other translator was a subject matter expert who guaranteed that language complexity and subject matter intricacies were not overlooked. One word was adjusted to be better culturally understood: “race” was translated into “ethnicity”. The final version, obtained from the two translations were matched, and when necessary, a discussion was made and then resolved with the original author (Fenn et al., 2020).

### Data collection

As per other validation procedures in the literature (Comrey and Lee, 1992; Anthoine et al., 2014), the number of samples presents a subject-to-item ratio of 1.

The study participants were students enrolled at Italian universities of healthcare professions, all recruited as volunteers.

A web survey was conducted between September 2020 and October 2020, following the guidelines provided by CHERRIES (Eysenbach, 2004; Ball Helen, 2019). The survey was shared through various social-based tools, such as university email address, SMS, and instant messaging within university degree course groups, to conduct enroll participants. To further reduce the potential bias in sample recruitment, each course director explained the importance of the research and encouraged all students to participate in the survey.

The following inclusion criteria were considered: (i) age over 18 years, (ii) ability to understand the Italian language (i.e., students from other countries temporarily in Italy through the ERASMUS program were excluded from the survey), (iii) be enrolled in a health profession degree program, (iv) to evaluate any random answer, based on another study (Bersani et al., 2022), one item of an attentional quality check (e.g., “Please score answer #5”) was proposed. The survey began with the acceptance of the informed consent.

The quiet ego is a non-defensive, growth-oriented identity that tends to resist the influence of immediate impulses and rewards. Moreover, the characteristics of the quiet ego have an indirect impact on wellbeing as they reflect a compassionate self-identity, involving an important aspect of self-transcendence and a sense of ecological identity focused on harmony and wellbeing derived from appreciating the reciprocal relationship between human activity and environmental health. Therefore, considering the findings from the literature published to date on the quiet ego, we reported some questions submitted to sample: “*Are you an only child?*” [yes or no]; “*Are both of your parents still alive?*” [yes or no]; “*Which degree program are you enrolled in*?”; “*Which year of the course are you in?*”; “*What was your first choice of degree program*?”. We wondered whether certain demographic characteristics or life experiences (such as being an only child vs. having siblings; experiencing the loss of one or both parents; choosing one degree course over another that exposes to experiences of compassion, etc.), could differentiate the expression of a quiet ego.

The survey was anonymous and completely voluntary, and no payment or other form of compensation was given to the participants.

### Ethical considerations

The Independent Institute Board of the Department of Human Neurosciences of Sapienza University of Rome has previously granted ethical approval for this study (N. Prot. 02/2020). The use of QES was granted by the principal author, Heydi Wayment. The participants were asked to sign an informed consent form before starting the survey, and the study data were kept confidential. Data collection and processing were conducted in accordance with privacy regulations.

### Statistical analysis

The analyses were carried out using SPSS, version 20.0 (SPSS, Inc., Chicago), and Jamovi, version 2.3.22. General information about the participants was obtained through descriptive analysis.

Demographic and psychological characteristics were compared using the analysis of variance (ANOVA) for continuous variables and the chi-squared test for categorical variables. The threshold for statistical significance was set at 0.05 (two-tailed, *p* < 0.05). The assumption of normality of data was verified.

A cluster analysis was conducted, and the subjects were grouped into two different age groups (20–25 and 26–42) to identify any differences in the quiet ego within the sample due to sociodemographic characteristics.

To assess the test-retest reliability, the test was given twice to the same participants at two different times. A smaller sample was used for the retest. The time interval between the two sessions was about a month, to avoid any potential learning effect (Fenn et al., 2020).

To evaluate the reliability of internal consistency, Cronbach's α coefficient was used. To evaluate the convergent and discriminant validity, correlation analyses were carried out between the iQES and the other measures observed in the Wayment et al.'s (2015a).

Before proceeding with factorial analyses to confirm the structure of the test, the Kaiser-Meyer-Olkin (KMO) value and Bartlett's test of sphericity were used.

The exploratory factor analysis (EFA) was used to examine the underlying factorial structure of the QES. Then, the confirmatory factor analysis (CFA) was carried out to confirm that underlying latent constructs exist and are related with the theory on the quiet ego. The following indices were used as descriptive measures of the overall model fit: root mean square error of approximation (RMSEA), root mean square residual (RMR), and standardized root mean square residual (SRMR) (Schermelleh-Engel et al., 2003).

To simplify data and minimize dimensionality, the main component analysis and the varimax rotation method were used, eigenvalues > 1 have been considered.

## Results

### Participant characteristics

A total of 160 questionnaires were completed. The survey sample included 160 young adults from Italy (131 female and 29 male students). The respondents were university students in healthcare professions aged between 20 and 42 years (M = 22.85; DS = 3.41).

According to the results of the survey and as shown by the number of students enrolled in the degree of health professions, most respondents were women (F = 81.9%). The main reason for this disparity is due to the lack of male students in this field. In Table 1, the socio-demographic characteristic of sample are described. In this sample, the QES scores have M = 52.5 and SD = 6.5. After conducting a cluster analysis, the sample was divided into two distinct age groups (20–25 years and 26–42 years) to better discern the differences in iQES between them.

**Table 1:** Sample descriptive statistics [binomial test, chi-square and Kolmogorov-Smirnov were used when appropriate; Sign = *p* < 0.5].

	***N***	**(%)**	***p***
Gender			**<0.000**
Male	29	18.1	
Female	131	81.9	
Age			**<0.000**
20–25	140	87.5	
26–42	20	12.5	
Only son			**<0.000**
Yes	18	11.2	
No	142	88.8	
Living parents			**<0.000**
Yes	154	96.2	
No	6	3.8	
Degree program			**<0.000**
Professional educator	24	15	
Physiotherapy	17	10.6	
Speech therapy	15	9.4	
Orthoptics	4	2.5	
Technician of psychiatric rehabilitation	47	29.4	
Occupational therapy	28	17.5	
Neuropsychomotor therapy evolutionary age	25	15.6	
Year of the course			**<0.000**
I	13	8.1	
II	44	27.5	
III	100	62.5	
Over	3	1.9	
First choice of degree program			0.236
Yes	88	55	
No	72	45	

N = 160. A significant p is in boldface type.

The results of the ANOVA indicated that the majority of the sample was women (81.9%) and notably young, with 87.5% aged between 20 and 25 years. Furthermore, only 11% of the sample are individuals who have no siblings, meaning they are an only child. Finally, 96% of them reported that both of their parents were alive. The sociodemographic characteristics or life experiences (i.e., being an only child vs. having siblings, experiencing the loss of one or both parents, etc.) did not show significant correlations with the QES (*p* > 0.05), and no statistically significant differences emerged between the two clusters.

### Reliability test

The reliability assessment was carried out through the verification of internal consistency and temporal stability. Internal consistency was evaluated using the Cronbach α coefficient and the split-half reliability coefficient, while temporal stability was evaluated through the test-retest reliability method.

A group of 37 students participated in a follow-up interview approximately 1 month later to evaluate the test-retest reliability. The correlation of the scores between the two evaluations was calculated, and the scores showed an acceptable temporal stability, *Pearson correlation* = 0.957; *Sig* <0.000 (optimal scores are > 0.90) (Tsang et al., 2017).

From the reliability analysis, an overall alpha coefficient of Cronbach of 0.76 and the split-half reliability coefficient of the global scale ≥ 0.70 were obtained. Both results confirm that the Italian version of the QES has a good internal consistency. An alpha range between 0.70 and 0.80 indicates high reliability.

The results indicate that, on average, there is a positive correlation among the items, with a mean correlation coefficient of 0.195 (for a broad construct, the optimal average inter-item correlation should fall within the range of 0.15–0.40). However, it is important to note that there is very little variability in these correlations, as indicated by a low variance of 0.26. This suggests that the items tend to be consistently correlated with each other across the sample, reflecting a stable pattern of association among the variables.

Furthermore, item-total correlation coefficients differ between 0.308 and 0.575, and they were all over 0.3, except for item 7 (0.214) that reported a low item-total correlation coefficient. Nevertheless, the 14 items were all integrated to the questionnaire.

The intra-class correlation coefficient has a value of F = 4.188; *p* ≤ 0.000 for both individual and average measurements, confirming a high level of confidence of the scale.

### Validity

To test concurrent criterion validity, we administered other different psychological scales as detailed previously. In this research, we only tested concurrent validity and did not test predictive validity. Pearson correlations between the iQES global score, Rosenberg Self-Esteem (RSE), Psychological Wellbeing (PWB) subscales, Subjective Happiness (SHS) global scores, and the CD-RISC 10 global score are shown in Table 2. All correlations are significant at the 0.01 level, except for the correlation with the SHS, which was significant at the 0.05 level.

**Table 2:** Pearson correlation between iQES and GOLD standard scales used for criterion validity.

	**iQES_tot**
RSE_TOT	0.375^**^
PWB_autonomy	0.379^**^
PWB_environmental_mastery	0.421^**^
PWB_personal_growth	0.463^**^
PWB_positive_relations	0.313^**^
PWB_Purpose_in_life	0.325^**^
PWB_Self_acceptance	0.347^**^
SHS	0.177^*^
CD_RISC_10	0.531^**^

^*^Correlation is significant at the 0.05 level (2-tailed).

^**^Correlation is significant at the 0.01 level (2-tailed).

RSE, Rosenberg Self-Esteem Scale; PWB, Psychological Wellbeing; SHS, Subjective Happiness Scale; CD_RISC_10, Connor-Davidson Resilience Scale_10.

### Construct validity

The EFA and CFA were used to test the construct validity. The calculation of the Kaiser-Meyer-Olkin value (KMO) and the Bartlett sphericity test confirmed the applicability of the factor analysis. A KMO value of 0.74 was obtained (values above 0.6 are considered sufficient to proceed with the PCA), while a *p* < 0.000 was obtained for the Bartlett's test of sphericity, which measures the congruence of the sample and supports that the model can be considered valid.

To extract common factors with eigenvalues > 1, the principal component analysis (PCA) and the varimax rotation method were used (Table 3). A good construct validity was confirmed by factor loadings for items > 0.40 and a cumulative variance contribution rate of > 60%.

**Table 3:** EFA of iQES.

**Matrix of the components rotated**				
	**Component**			
	**1**	**2**	**3**	**4**
QES_9	**0.824**	−0.038	0.064	0.060
QES_5	**0.804**	0.137	0.182	0.152
QES_14	**0.615**	−0.148	0.354	−0.027
QES_1	**0.577**	0.177	−0.011	0.192
QES_4	**0.489**	0.415	0.088	0.335
QES_7	−0.047	**0.845**	−0.071	−0.087
QES_3	0.090	**0.840**	0.047	0.039
QES_12	0.079	**0.691**	−0.054	0.230
QES_10	0.102	−0.017	**0.858**	0.053
QES_2	0.050	0.040	**0.806**	0.135
QES_6	0.207	−0.058	**0.754**	0.067
QES_13	0.008	0.067	0.055	**0.810**
QES_11	0.196	−0.024	0.157	**0.629**
QES_8	0.269	0.383	0.028	**0.510**

Extraction Method: Principal component analysis.

Rotation Method: Varimax with Kaiser normalization. Loadings with absolute values of 0.40 or more are shown in boldface type.

**Figure 1:**
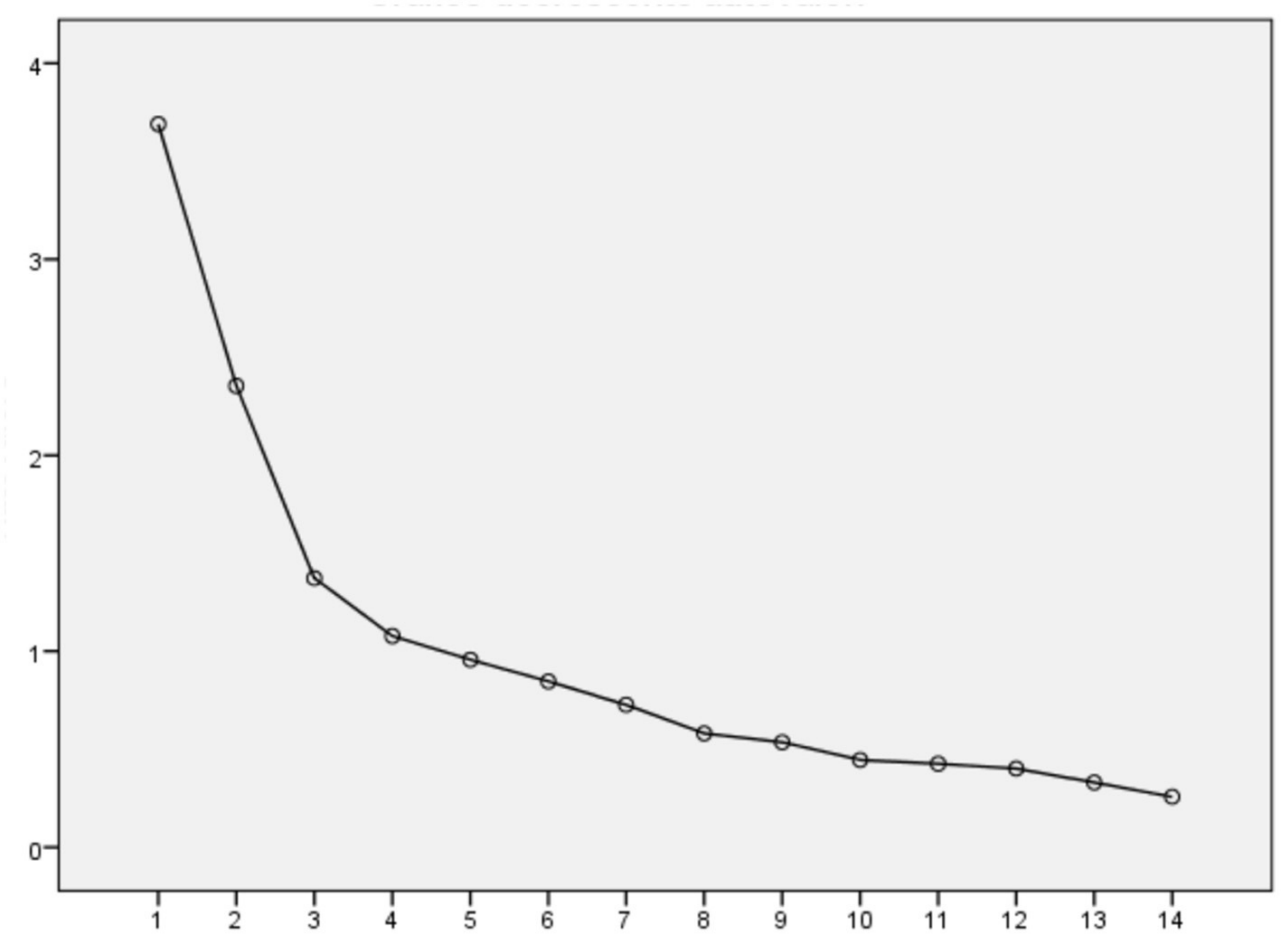
Scree plot of eigenvalue.

The main goal of the EFA was to reduce the number of variables required to describe the data derived from the iQES and to explore the dimensions underlying its variables. Figure 1 shows the scree plot of eigenvalues, where a first predominant factor emerges, followed by a second, a third, and a fourth important factor, although much less strong than the first, and then a substantial flattening of the curve is observed starting from the fifth factor.

In accordance with the main author theory (Wayment et al., 2015a), a four-factor solution emerged, confirming the domains of the QES structure: detached awareness, perspective-taking, inclusive identity, and personal growth.

All the items concur to estimate the same construct of the quiet ego with a percentage of explained variance that is 60.6%.

### Confirmative factor analysis

To test the fit capacity of iQES, the confirmative factor analysis was performed.

As the χ2-test is sensitive both to the sample size and the violation of the multivariate normality assumption, it cannot be used as the unique index for judging the model fit, and it is necessary to associate it to other fit indices (Schermelleh-Engel et al., 2003).

In addition, Kline (2015) recommended the evaluation of *root mean square error of approximation* (RMSEA), *standardized root mean square residual* (SRMR), and *comparative fit index* (CFI). The χ^2^-test, RMSEA, and SRMR are absolute fit indices, and they are used to assess the extent to which the model in question fits perfectly, and a larger value represents a poorer fit.

The results showed a χ^2^-value of 120 (df = 71); *p* < 0.001 [χ^2^/df= 1.7 (“ <2” indicates a good model fit)]; the RMSEA= 0.0656 is very good (acceptability is <0.10); SRMR= 0.0663 (acceptability is <0.05); the CFI= 0.916 indicates a good fit (Schermelleh-Engel et al., 2003).

**Figure 2:**
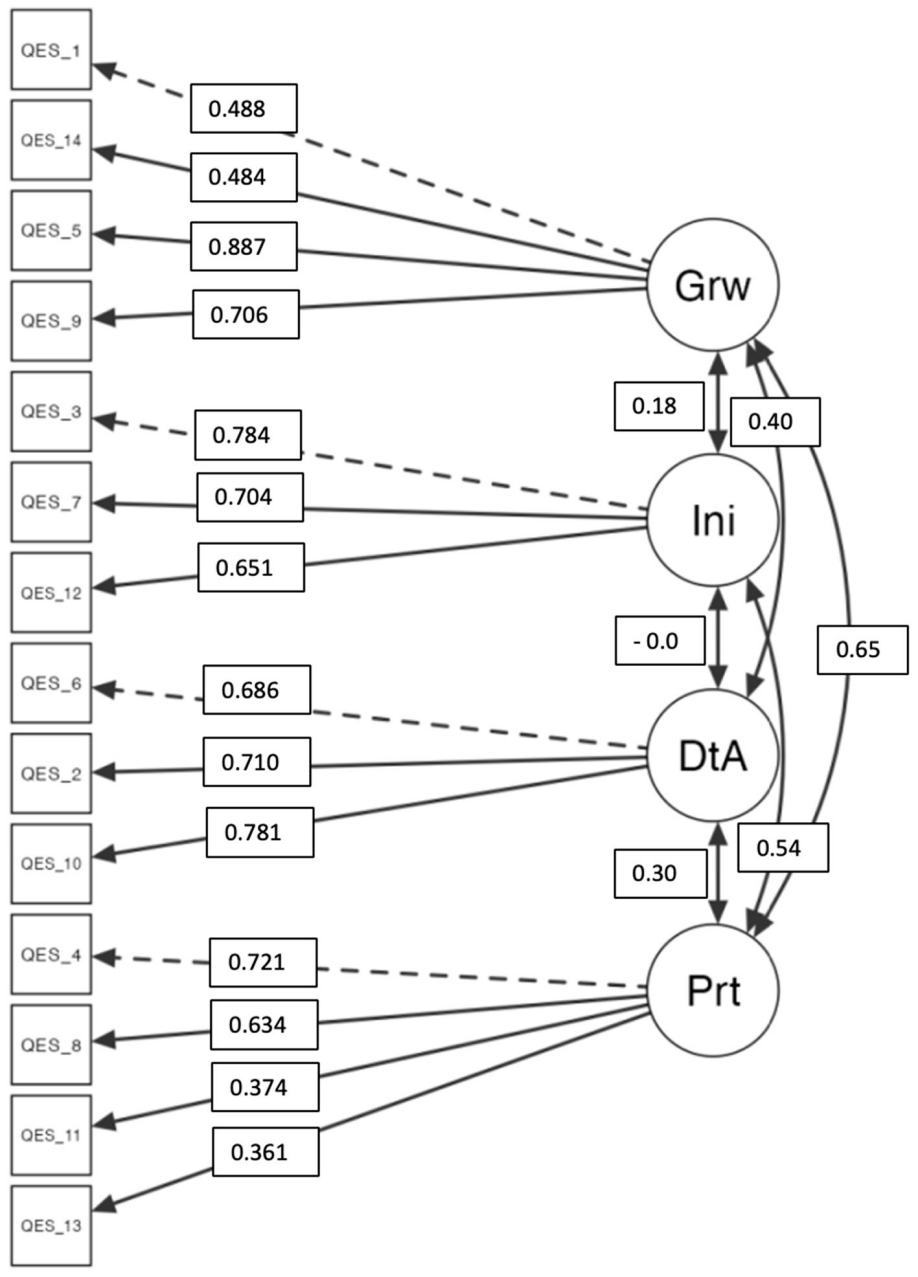
Flowchart of CFA. Grw, Growth; Ini, Inclusive Identity; DtA, Detached Awareness; PrT, Perspective taking. The structural configuration of the iQES with standardized factor loadings derived from CFA. All factor loadings demonstrated significance at *p* < 0.001.

The results confirm the goodness of the model (Figure 2).

## Discussion

To the best of our knowledge, this is the first study of translation, reliability, and validity of the Quiet Ego Scale in a sample of Italian students (Table 4). In the validation process of the Italian version of QES, we carefully followed the methods outlined in the literature and the most significant international guidelines (Beaton et al., 2000; International Test Commission, 2017; Tsang et al., 2017; Fenn et al., 2020). According to the guidelines for assessing goodness-of-fit indices proposed by Schermelleh-Engel et al. (2003), the statistical analysis has demonstrated that the scale exhibits a good structural validity and that it is reliable and effective at measuring the concept of the quiet ego.

**Table 4:** Quiet Ego Scale (iQES).

**1**	***Penso che sia importante vivere nuove esperienze che ti sfidano a pensare in modo diverso a te stesso e al mondo***
	I think it is important to have new experiences that challenge how you think about yourself and the world
**2**	Mi ritrovo a fare le cose senza prestare molta attenzione^*^
	I find myself doing things without paying much attention^*^
**3**	*Mi sento in connessione con tutti gli esseri viventi*
	I feel a connection to all living things
**4**	*Prima di criticare qualcuno provo a immaginare come mi sentirei se fossi al suo posto*
	Before criticizing somebody, I try to imagine how I would feel if I were in their place
**5**	*Per me la vita è stata un continuo processo di apprendimento, cambiamento e crescita*
	For me, life has been a continuous process of learning, changing, and growth
**6**	Svolgo lavori o compiti automaticamente, senza essere consapevole di ciò che sto facendo^*^
	I do jobs or tasks automatically, without being aware of what I'm doing^*^
**7**	*Mi sento in connessione con gli estranei*
	I feel a connection with strangers
**8**	*Quando sono arrabbiato con qualcuno, in genere provo a mettermi nei suoi panni per un po'*
	When I'm upset at someone, I usually try to put myself in his or her shoes for a while
**9**	*Ho la sensazione di essermi accresciuto molto come persona nel corso del tempo*
	I have the sense that I have developed a lot as a person over time
**10**	Mi sbrigo a fare le cose senza prestarci molta attenzione^*^
	I rush through activities without being really attentive to them^*^
**11**	A volte trovo difficile vedere le cose dal punto di vista di un'altra persona^*^
	I sometimes find it difficult to see things from another person's point of view^*^
**12**	*Mi sento in connessione con persone di altre etnie*
	I feel a connection to people of other races
**13**	*Cerco di prendere in considerazione l'opinione di tutti in caso di disaccordo prima di prendere una decisione*
	I try to look at everybody's side of a disagreement before I make a decision
**14**	Quando ci penso, non sono realmente migliorato come persona nel corso degli anni^*^
	When I think about it, I haven't really improved much as a person over the years^*^

^*^Reverse-coded item.

The results demonstrated the good psychometric properties of the iQES in terms of assessing the quiet ego construct. It can be used to measure some personality traits, as the quiet ego disposition, in specific way.

The QES represents a self-determined identity of self, which despite being oriented to psychological growth is confronted with the other in an altruistic and less selfish way.

In the present study the Quiet Ego was positively related to self-esteem, psychological wellbeing, subjective happiness, resilience, supporting prior studies (Wayment et al., 2015a; Akca and Sumer, 2016; Watson, 2018; Bistricky et al., 2022).

A recent study of Wayment et al. (2019) suggested, in fact, the possibility of ameliorating self-regulatory abilities through a therapeutic approach based on the quiet ego to better manage stressful situations.

From another field of research, Liu et al. (2022) found that, in a group of subjects, quiet ego traits can be improved, thanks to the application of a brief training on quiet ego's contemplation. In this case, the quiet ego had shown to be related with flourishing, an extensive concept of psychological wellbeing, and that both were sustained by emotional intelligence abilities.

In a previous study, Kaufman et al. (2019) demonstrated that the quiet ego is negatively related to the dark triad of personality, suggesting a possible role of the quiet ego in the recent field of research on narcissism called “Dark Triad” of personality (Muris et al., 2017).

In this regard, the results also suggest that the quiet ego may be linked to specific abilities that would allow an individual to be aware and be able to see other points of view in addition to their own. For example, it might include the ability to use functional emotional regulation strategies that allow individuals to think and be less hostile and aggressive, and/or to act cooperatively (Wayment and Cavolo, 2019).

Another specific field where the concept of the quiet ego can be applied is in working with health professionals.

In fact, health workers, especially those employed in emergency departments, or those in wards with high emotional intensity (oncology unit, intensive care unit, etc.), may be more exposed to stressful conditions and experience high levels of emotional distress. In these cases, a psychological support can be aimed to reduce compassion fatigue and ameliorate emotion regulation skills (Wayment et al., 2019).

The expression “quiet ego” aligns with the principles in mindfulness and compassion-focused therapy, presenting a potentially impactful synergy for fostering mental health-supportive lifestyles and attitudes. In fact, mindfulness entails a non-judgmental, attentive awareness of the present moment. A “quiet ego” represents an approach to the world characterized by acceptance, devoid of excessive self-centeredness or ego-centricity (Wayment et al., 2011). This alignment enhances self-awareness within oneself and the broader context. On the other hand, compassion-focused therapy centers around cultivating compassionate self-acceptance, extending this compassion to oneself and others. The concept of a “quiet ego” harmonizes with this practice by mitigating self-centeredness and creating room for a compassionate stance toward both self and others.

Adopting a “quiet ego” approach contributes to mental health promotion by mitigating stress and ego-related anxiety, fostering emotional resilience (Phillips and Ferguson, 2013). Self-awareness and compassion emerge as pivotal resources for navigating the challenges and stressors of daily life (Bauer and Weatherbie, 2023).

Mindful awareness of the present moment encourages a more balanced lifestyle, steering individuals toward deliberate and health-conscious choices (Zessin et al., 2015). A “quiet ego” can play a supportive role in stress management and decision-making oriented toward the overall wellbeing.

Diminishing self-centeredness and promoting compassion positively influence attitudes toward oneself and others. This positive shift contributes to the cultivation of healthier, more gratifying relationships and stands as a fundamental element in maintaining a positive mental outlook.

Integrating concepts such as the “quiet ego,” mindfulness, and compassion crafts a comprehensive approach to mental health (Raab, 2014). This amalgamation proves applicable in daily personal practices and as an integral component of therapeutic approaches, offering support for psychological wellbeing and facilitating personal growth (Watson, 2018).

The concepts of quiet ego can be implemented in numerous other domains where personal and others' wellbeing, as well as personal growth, are objectives to achieve. For instance, within organizational contexts, a quiet ego may foster a more collaborative, compassionate, and psychologically well-oriented work environment, contributing to reducing stress and enhancing the overall job satisfaction. A team leader with a greater quiet ego is more likely to be empathetic, attentive, and oriented toward personal and organizational growth, thereby promoting a climate of trust, mutual respect, and collaboration within the group (Buonomo et al., 2021).

A work environment that encourages the quiet ego can facilitate open sharing of ideas, collaboration, and innovation; indeed, individuals with a quiet ego are more prone to listening to others' perspectives and be open to new ideas, which can stimulate creativity and organizational innovation (Gilbert et al., 2022). Finally, promoting the development of the quiet ego among employees can foster personal and professional growth (Wayment et al., 2018; Buonomo et al., 2021).

In the political field, political leaders with a quiet ego tend to be more oriented toward public service, less prone to selfishness, and more likely to make decisions based on the common good rather than personal interests (Chew and Ang, 2023). Individuals with a quiet ego are more inclined to manage conflicts constructively, seeking solutions that meet the diverse needs of the involved parties without being influenced by selfishness or the pursuit of personal power, thereby fostering citizens' trust and political stability. Individuals with a quiet ego are more likely to set aside personal differences to work together toward common goals, promoting dialogue and compromise, further fostering collaboration among political parties and diverse ideologies (Watson, 2018; Al-Kire et al., 2022).

In summary, quiet ego is positively associated with a personal development measure that includes not only functional self-regulation skills, self-compassion, assertive and non-aggressive communication skills, and cooperative and growth-oriented social competence but also a spiritual sense of self-transcendence. All these can be considered the protective factors because they can be associated with increased stress management skills and functional coping strategies (Kesebir, 2014; Liu et al., 2022).

Our findings add to a growing body of literature supporting the validity of the iQES and the multidimensional nature of the quiet ego construct.

Further studies will be needed to understand the possible role of the quiet ego in increasing individual resources and possible uses in psychological treatments, although some studies on small samples have already been carried out, yielding results of considerable interest (Wayment et al., 2015b, 2019).

### Limitations

In light of the study's results, it is necessary to consider several limitations. First, the quiet ego is a new and minimally researched personality trait. Indeed, the quiet ego characterizes a different conception of the “ego,” where attention is directed toward both one and others, and for these reasons, it has been studied not only in positive psychology but also in different and emerging fields such as business (Gilbert et al., 2022), human mating (Lucchi Basili and Sacco, 2020), work engagement (Buonomo et al., 2021), among others.

Second, the study population consists solely of university students and does not fully represent Italian culture. Since research indicates that, at the core of the quiet ego, there is a tendency toward psychosocial maturation that supports the emergence of a more integrated, stable, and consistent self-structure. In the present study, it was hypothesized that students in healthcare professions, by choosing a helping profession, may exhibit a greater predisposition toward personal growth, compassion, inclusive identity, and self-determination, characteristics that describe a quiet ego.

Third, although the questionnaire was structured by inserting an attention control item similar to that in the study by Bersani et al. (2022), it is necessary to consider various aspects regarding the completion of the survey: (i) it could be completed at any place, increasing the probability of making mistakes or paying little attention; (ii) it could be completed at any time, making it more likely here as well the possibility of providing responses that may have been influenced by different moods.

Fourth, another limitation concerns the availability of freely validated assessment tools in Italian. This aspect is crucial as research necessitates access to suitable assessment instruments in the language of the study sample, preferably at no cost.

Fifth, in the second experiment of Wayment et al.'s (2015a) study, the QES value was not correlated with the participants' age, gender, or ethnicity, while in the third experiment of Wayment et al.'s (2015a) study, the QES value was higher in women. To date, no specific studies have been conducted to fully understand the nature of any substantial differences in the quiet ego attributable to gender. Therefore, it is not possible to reach clear conclusions. However, it appears essential to attempt to understand the presence of any differences.

Moreover, as the literature on this topic is still limited, it was challenging to develop a broader discussion. However, our results highlight the novelty and the interest that characterizes the quiet ego as well as the readability of the study.

## Data availability statement

The raw data supporting the conclusions of this article will be made available by the authors, without undue reservation.

## Ethics statement

The studies involving humans were approved by the Independent Institute Board of the Department of Human Neurosciences of Sapienza University of Rome (N. Prot. 02/2020). The studies were conducted in accordance with the local legislation and institutional requirements. The participants provided their written informed consent to participate in this study.

## Author contributions

LB: Conceptualization, Formal analysis, Methodology, Writing—original draft, Writing—review and editing. CS: Data curation, Formal analysis, Writing—review and editing. LG: Data curation, Resources, Writing—original draft. VP: Supervision, Writing—review and editing. FR: Data curation, Writing—original draft. GM: Data curation, Writing—original draft. DV: Conceptualization, Resources, Writing—original draft. EP: Conceptualization, Supervision, Writing—original draft, Writing—review and editing.
